# New Translational Research Provides Insights into Liver Dysfunction in Sepsis

**DOI:** 10.1371/journal.pmed.1001341

**Published:** 2012-11-13

**Authors:** John C. Marshall

**Affiliations:** Departments of Surgery and Critical Care Medicine, and the Keenan Research Centre of the Li Ka Shing Knowledge Institute, St. Michael's Hospital, University of Toronto, Toronto, Ontario, Canada

## Abstract

John Marshall discusses new research detailing the early onset in liver dysfunction in a rodent sepsis model, which he says can fundamentally change our understanding of this common clinical problem.

**Linked Research Article**

This Perspective discusses the following new study published in *PLOS Medicine*:

Recknagel P, Gonnert FA, Westermann M, Lambeck S, Lupp A, et al. (2012) Liver Dysfunction and Phosphatidylinositol-3-Kinase Signalling in Early Sepsis: Experimental Studies in Rodent Models of Peritonitis. PLoS Med 9(11): e1001338. doi:10.1371/journal.pmed.1001338

Experimental studies in a rat model of faecal peritonitis conducted by Michael Bauer and colleagues show that in this model, changes in liver function occur early in the development of sepsis, with potential implications for prognosis and development of new therapeutic approaches.

Success in the support of critically ill patients comes at a cost. The emergence of intensive care units (ICUs) a half century ago led to the unprecedented survival of patients who previously would have died from severe infection, traumatic injuries, or lethal organ system insufficiency [1]. This success, however, spawned a spectrum of new clinical disorders amongst the critically ill—acute respiratory distress syndrome, septic shock, acute renal failure—that only evolved because of the survival of patients who, without intervention, would have died [2]. Collectively, these potentially lethal abnormalities of vital organ function are known as multiple organ dysfunction syndrome (MODS), a designation that reflects both the importance of deranged organ interactions and our basic ignorance in understanding them [3].

Liver dysfunction as a consequence of life-threatening battlefield injury was first described by Bywaters in 1946 [4]. But it was only with the widespread establishment of ICUs in the 1960s that jaundice and liver dysfunction became a common manifestation of critical illness [5],[6]. Descriptive reports suggested that the causes were multiple—transfusion, parenteral nutrition, infection, splanchnic ischemia, hepatotoxic medications—but both the pathogenesis and the clinical consequences were obscure. Despite the critical role played by the liver in normal metabolic and immunologic homeostasis, its role in the expression of MODS has been largely unknown.

In a tour de force of translational investigation carried out by Michael Bauer and colleagues and published in this issue of *PLOS Medicine*, multiple sources of data—from experimental work in isolated cell cultures and genetically modified mice to studies carried out in critically ill patients—were used to probe the mechanisms and consequences of liver dysfunction in sepsis [7]. These findings can fundamentally change our understanding of this common, but clinically occult, problem.

## Hepatocellular Dysfunction Is Common in Sepsis, and Involves Impaired Excretion of Biotransformed Compounds

Most descriptions of liver dysfunction in critical illness focus on jaundice and increased circulating levels of bilirubin [8]. In contrast, Bauer and colleagues show that levels of both conjugated and unconjugated chenodeoxycholic and taurodeoxycholic acid are increased in septic patients on the day of diagnosis, and that these increased levels show a stronger correlation with 28-day mortality than do bilirubin levels. Analysis of transcriptional changes in liver tissue from septic rats revealed reduced expression of pathways involved in amino acid and fatty acid metabolism; impairment of processes involved in the biotransformation of both endogenous substrates and medications were specifically associated with an increased risk of death. Consistent with these changes in transcription, the most severely ill septic animals demonstrated extensive cholestasis, and impaired excretion of indocyanine green, a dye whose clearance requires hepatocellular processing. The authors show that liver dysfunction in lethal rat sepsis occurs in the absence of evidence of hepatocellular injury, suggesting that the changes in the liver are a consequence of alterations in gene transcription, rather than non-specific hepatocellular injury. They further found reduced sinusoidal perfusion and depletion of ATP content, pointing to alterations in energy availability as a mechanism of the observed changes.

## Phosphatidylinositol-3 (PI3) Kinase Signaling Contributes to Altered Transporter Expression and Localization in Sepsis

Finally, in an effort to identify a modifiable target for the functional hepatocellular derangements of sepsis, the authors used a combination of pharmacologic and molecular approaches to assess the role of PI3 kinase, a key signaling enzyme involved in both transporter trafficking and inflammatory gene expression. Mice deficient for the PI3Kγ gene showed preserved morphology and function of the bile canaliculi, suggesting that the activation of PI3 kinase contributes to the functional alterations seen in sepsis. Intriguingly, neutrophil recruitment into the liver was reduced in PI3Kγ^−/−^ mice, raising the possibility that circulating neutrophils contribute to the hepatic lesion of sepsis.

## Clinical Implications: A Role for the Liver in Early Sepsis

In aggregate, a series of complementary studies create a compelling description of liver dysfunction in sepsis, and identify activation of PI3 kinase–dependent signalling as a pathogenetic mechanism. However, there are limitations. Liver dysfunction in human sepsis is driven not only by the biologic changes directly resulting from sepsis, but also by the consequences of multiple supportive interventions—for example, medications that are directly hepatotoxic or alter liver blood flow, and the mode of nutritional support. Moreover, fecal peritonitis in a rat is a poor model for the complex process that occurs in humans [9]. Rat models are provided with minimal physiologic support and use of antibiotics or source control for the originating infection. In contrast, human sepsis most commonly occurs in association with pneumonia, typically affects elderly patients with significant pre-existing co-morbid conditions, and is highly associated with a genetic predisposition, resulting from single nucleotide polymorphisms in a wide array of genes involved in the innate immune response [10]. Thus, it is not surprising that alterations in bile acid levels seen in humans involved both conjugated and unconjugated acids, suggesting changes that are more complex than the simple changes seen in a rat model simple loss of the conjugating machinery of the hepatocytes.

Globally, sepsis is a leading cause of potentially preventable mortality and morbidity, accounting for high levels of hospitalizations in countries such as the United States [11] and Brazil [12]. While studies in humans are the ultimate testing ground for mechanistic hypotheses that can give rise to new treatments, interventional studies are costly and methodologically challenging. Thus, the hybrid translational model embodied in the work reported here by Bauer and colleagues not only provides a valuable new insight into the pathogenesis of liver derangements in sepsis, but even more importantly, establishes a model that should be welcomed and embraced by scientists working in the field.

## Abbreviations

ICU: intensive care unit
MODS: multiple organ dysfunction syndrome
PI3: phosphatidylinositol-3
